# Mice Fed an Obesogenic Western Diet, Administered Antibiotics, and Subjected to a Sterile Surgical Procedure Develop Lethal Septicemia with Multidrug-Resistant Pathobionts

**DOI:** 10.1128/mBio.00903-19

**Published:** 2019-07-30

**Authors:** Sanjiv K. Hyoju, Alexander Zaborin, Robert Keskey, Anukriti Sharma, Wyatt Arnold, Fons van den Berg, Sangman M. Kim, Neil Gottel, Cindy Bethel, Angella Charnot-Katsikas, Peng Jianxin, Carleen Adriaansens, Emily Papazian, Jack A. Gilbert, Olga Zaborina, John C. Alverdy

**Affiliations:** a Department of Surgery, University of Chicago, Chicago, Illinois, USA; b Amsterdam UMC, University of Amsterdam, Amsterdam, The Netherlands; c Department of Medicine, University of Chicago, Chicago, Illinois, USA; d Department of Pathology, University of Chicago, Chicago, Illinois, USA; e Guangdong Province Hospital of Chinese Medicine, China; f Department of Surgery, Radboud University Medical Center, Nijmegen, The Netherlands; Yale School of Public Health

**Keywords:** Western diet, gut microbiome, gut-derived sepsis, pathobionts, surgery

## Abstract

Despite antibiotics and sterile technique, postoperative infections remain a real and present danger to patients. Recent estimates suggest that 50% of the pathogens associated with postoperative infections have become resistant to the standard antibiotics used for prophylaxis. Risk factors identified in such cases include obesity and antibiotic exposure. To study the combined effect of obesity and antibiotic exposure on postoperative infection, mice were allowed to gain weight on an obesogenic Western-type diet (WD), administered antibiotics and then subjected to an otherwise recoverable sterile surgical injury (30% hepatectomy). The feeding of a WD alone resulted in a major imbalance of the cecal microbiota characterized by a decrease in diversity, loss of *Bacteroidetes*, a bloom in *Proteobacteria*, and the emergence of antibiotic-resistant organisms among the cecal microbiota. When WD-fed mice were administered antibiotics and subjected to 30% liver resection, lethal sepsis, characterized by multiple-organ damage, developed. Notable was the emergence and systemic dissemination of multidrug-resistant (MDR) pathobionts, including carbapenem-resistant, extended-spectrum β-lactamase-producing Serratia marcescens, which expressed a virulent and immunosuppressive phenotype. Analysis of the distribution of exact sequence variants belonging to the genus *Serratia* suggested that these strains originated from the cecal mucosa. No mortality or MDR pathogens were observed in identically treated mice fed a standard chow diet. Taken together, these results suggest that consumption of a Western diet and exposure to certain antibiotics may predispose to life-threating postoperative infection associated with MDR organisms present among the gut microbiota.

## Abstract (precis)

Obesity remains a prevalent and independent risk factor for life-threatening infection following major surgery. Here, we demonstrate that when mice are fed an obesogenic Western diet (WD), they become susceptible to lethal sepsis with multiple organ damage after exposure to antibiotics and an otherwise-recoverable surgical injury. Analysis of the gut microbiota in this model demonstrates that WD alone leads to loss of *Bacteroidetes*, a bloom of *Proteobacteria*, and evidence of antibiotic resistance development even before antibiotics are administered. After antibiotics and surgery, lethal sepsis with organ damage developed in in mice fed a WD with the appearance of multidrug-resistant pathogens in the liver, spleen, and blood. The importance of these findings lies in exposing how the selective pressures of diet, antibiotic exposure, and surgical injury can converge on the microbiome, resulting in lethal sepsis and organ damage without the introduction of an exogenous pathogen.

## INTRODUCTION

The most common, costly, and deadly complication after major surgery is infection (1–3). Despite the use of broader and more frequent dosing of antibiotics, surgical infections continue to occur at unacceptable rates. Recent data indicate that 50% of all surgical-site infections are now due to organisms that have become resistant to the standard antibiotics used for prophylaxis (4, 5). Although the precise source of these pathogens is unknown, in humans, the gut has been identified as a major reservoir for antibiotic-resistant bacteria (6, 7). Precisely how pathogenic organisms in the gut might contribute to postoperative infection, however, remains unknown.

The microbiome a given patient harbors prior to undergoing major surgery is unknown and is likely to be affected not only by diet but also by recent antibiotic exposure, travel history, and the diagnosis of the underlying disease for which surgery is being performed, such as cancer (8, 9). Patients are often exposed to multiple and prolonged antibiotics prior to high-risk surgery when they undergo biopsies, radiation, or chemotherapy or present with an infection. The extent to which these exposures alter the intestinal microbiome and the influence of the altered microbiome on postoperative infection are largely unknown (10). Compounding these factors is the observation that more than 30% of patients undergoing major surgery in the United States are obese (11), which itself is an independent risk factor for the development of all postoperative infections (12, 13). In the present study, we hypothesized that an obesogenic diet, combined with antibiotic exposure, would predispose a host to infection-related morbidity following a major surgical intervention via effects that involve the intestinal microbiome. Therefore, the aims of the present study were to (i) create a novel mouse model of lethal infection in which mice are fed an obesogenic diet, are administered antibiotics, and undergo an otherwise recoverable sterile surgical injury, and (ii) define the microbiota and pathobionts that are associated with lethal infection in this model and determine their source, antibiotic resistance patterns, and effect on immune cell function. Results indicate that an obesogenic diet and antibiotic exposure impose selective pressure on the intestinal microbiota such that lethal infection develops following an otherwise-recoverable surgical injury. As far as we are aware, this is the first animal model to demonstrate spontaneous emergence and dissemination of virulent, drug-resistant pathobionts with multiple-organ failure and lethal sepsis without the introduction of an exogenous pathogen.

## RESULTS

### The cecal microbiota of Western diet-fed mice demonstrates loss of bacteroidetes, a bloom in proteobacteria, and antibiotic resistance.

**FIG 1:**
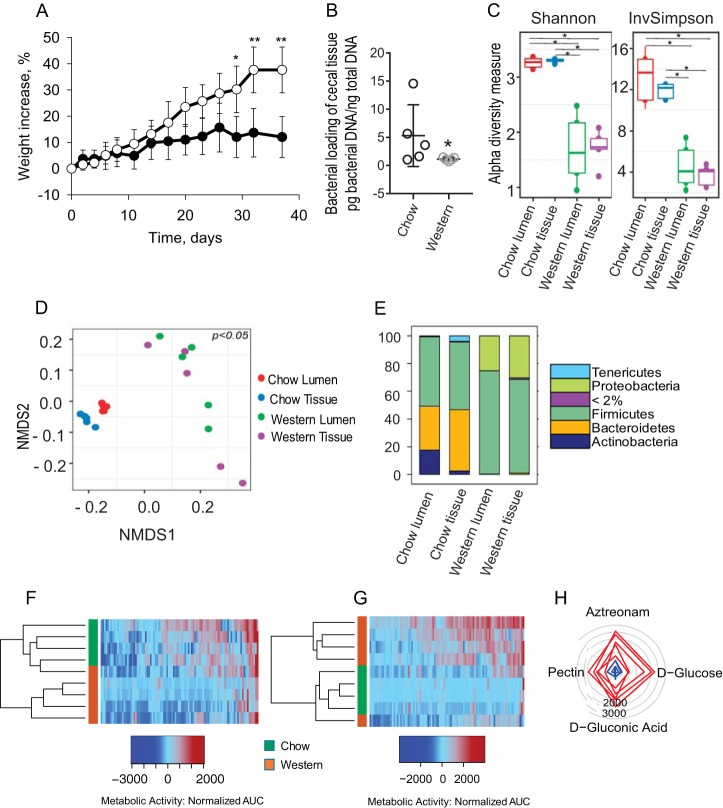
Role of Western diet on gut microbial structure, membership, and function. (A) Change in mouse weight (%) over time: Western diet (open circles) versus chow (solid circles). *, *P* < 0.0001 (chow, *n* = 4; Western diet, *n* = 5). Statistical bars depict the standard errors (SEM). (B) Total bacterial DNA concentration in cecal tissue as determined by qPCR (*n* = 5 per group, *P* = 0.037 [nonparametric Mann-Whitney test]). (C) Alpha diversity based on Shannon and inverse Simpson (invSimpson) indexes. Asterisks indicate statistical significant differences (*n* = 5 mice per group; *, *P* < 0.05). (D) Nonmetric multidimensional scaling (NMDS) plots based on weighted UniFrac dissimilarity matrix between the four groups (*n* = 5 mice per group). The multigroup PERMANOVA revealed significant differences between groups: chow lumen versus chow tissue (*P* = 0.01); chow tissue versus Western tissue (*P* = 0.002); and chow lumen versus Western lumen (*P* = 0.004). There was no significant difference for Western lumen versus Western tissue. (E) Stack plots showing distribution of phyla with a relative abundance of >2%. The taxa with <2% abundance are placed under one category, i.e., “<2%.” Statistical analysis tested for the differential abundance at a *P* value of <0.05 with the Benjamini-Hochberg FDR correction in ANCOM (*n* = 5 mice per group). (F and G) Heat maps of phenotype microarrays tested with GENIII plate under anaerobic (F) and aerobic (G) conditions (chow, *n* = 4; Western diet, *n* = 5). (H) Radial plot representing the set of substrates with significantly higher metabolic activity of microbial populations from Western diet-fed versus chow-fed mice. Radial numbers represent the AUC values. Each line on the plot represents an individual mouse. The degree to which the line deviates toward a given metabolite on the plot represents the predisposition of the bacterial community to metabolize that metabolite.

Mice were fed a high-fat/low-fiber Western diet (WD) diet versus chow for 40 days. WD-fed mice displayed a significant increase in weight compared to chow-fed mice (*n* = 4 for chow group and *n* = 5 per Western group; *, *P* < 0.001 [*t* test]) (Fig. 1A) with typical fat deposition in the liver. Serum C-reactive protein (CRP), interleukin-6 (IL-6), and body temperature (see Fig. S1A to C in the supplemental material) trended higher in WD-fed mice with the rare detection of positive cultures within the blood, liver, and spleen (see Fig. S1D in the supplemental material). A significant alteration in cecal microbiota composition and function was observed (Fig. 1B to E). A decrease in total bacterial density in the cecal tissue-associated microbiota was observed in WD-fed mice by quantitative PCR (qPCR) (Fig. 1B), as well as a significant decrease in alpha diversity in both the lumen and tissue-associated microbiota, as measured by Shannon and inverse Simpson indexes (Fig. 1C). Microbiota composition was significantly different between WD- and chow-fed mice in both the lumen and tissue compartments (cecum) based on a weighted UniFrac metric (Fig. 1D). In the lumen, the *Bacteroidetes*/*Firmicutes*/*Proteobacteria* (B:F:P) ratio was 16:25:0 in chow-fed mice versus 1:375:125 in WD-fed mice. In the tissues, the B:F:P ratio was 220:245:3 in chow-fed mice versus 9:680:300 in WD-fed mice (Fig. 1E). Compositional changes in the microbiota were accompanied by functional changes, as judged by phenotypic microarray analysis performed under anaerobic (Fig. 1F) and aerobic (Fig. 1G) conditions. Under anaerobic conditions, microbiota in WD-fed mice displayed a lower metabolic activity pattern compared to chow-fed mice (Fig. 1F). The opposite effect was observed under aerobic conditions (Fig. 1G). The low metabolic activity of the microbiota under anaerobic conditions in WD mice was consistent with the loss of *Bacteroidetes*, whereas high metabolic activity under aerobic conditions was consistent with the observed bloom in *Proteobacteria*. A higher saccharolytic potential was observed in the microbiota of WD-fed mice based on a significant increase in glucose and gluconic acid consumption. In addition, the antibiotic resistance potential observed in the microbiota of WD-fed mice was striking, as judged by their significant growth in the presence of aztreonam (Fig. 1H). Analysis of the entire carbohydrate set on the GENIII plates demonstrated a clear pattern of a preference of multiple carbohydrate consumption under aerobic conditions among the microbiota of WD-fed mice (see Fig. S2 in the supplemental material).

10.1128/mBio.00903-19.1 FIG S1 Analysis of sepsis markers in WD- and chow-fed mice measured before antibiotic treatment. (A) C-reactive protein (CRP). *P* = NS (not significant; *n* = 4 per group). (B) Serum IL-6 levels in pg/ml. *P* = NS (*n* = 4 per group). (C) Body surface temperature measured by laser instrument reflecting minimum and maximum measurements. *P* = NS (*n* = 5 mice per group). (D) Colonization of blood, liver, and spleen by Gram-negative bacteria on MacConkey plates. *P* = NS (*n* = 5 mice per group; Mann-Whitney test). Download FIG S1, PDF file, 0.03 MB. Copyright © 2019 Hyoju et al. 2019 Hyoju et al. This content is distributed under the terms of the Creative Commons Attribution 4.0 International license.

10.1128/mBio.00903-19.2 FIG S2 Comparative carbohydrate metabolic analysis of cecal contents isolated from WD- and chow-fed mice. Analysis using GENIII plates to assess microbial phenotype. Download FIG S2, PDF file, 0.1 MB. Copyright © 2019 Hyoju et al. 2019 Hyoju et al. This content is distributed under the terms of the Creative Commons Attribution 4.0 International license.

### WD-fed mice exposed to antibiotics develop lethal sepsis following an otherwise recoverable and sterile surgical injury.

**FIG 2:**
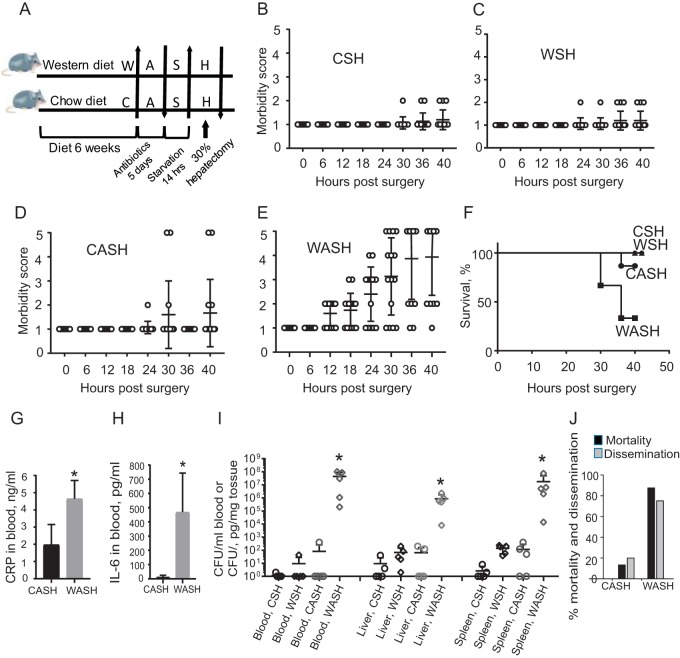
Effect of Western diet (W) on lethal sepsis following antibiotic exposure (A), short-term starvation (S), and a 30% hepatectomy (H) (WASH). (A) Experimental protocol. (B to E) Postoperative morbidity scores in CSH (chow + starvation + hepatectomy), CASH (chow + antibiotics + starvation + hepatectomy), WSH (Western diet + starvation + hepatectomy), and WASH (Western diet + antibiotics + starvation + hepatectomy). Morbidity scores (MS): MS1, healthy mice; MS2, ruffled fur, normal fecal pellets (three to four pellets over 24 h); MS3, ruffled fur, fewer fecal pellets (one to two pellets over 24 h), hunched posture, increased respirations; MS4, ruffled fur, no pellets, hunched posture, increased respirations, do not move to touch; and MS5, animal on side, minimally responsive, rapid shallow respirations, gasping, moribund. WASH treatment compared to all groups: *P* < 0.0001 at 18, 24, 30, 36, and 40 h (*n* = 15/group [two-way ANOVA]). (F) Kaplan-Meier survival curves. WASH treatment compared to all other groups: *P* < 0.0001 (*n* = 15 mice/group, log-rank [Mantel-Cox] test). (G) Serum CRP for CASH and WASH treatments in blood samples collected at POD2 (*n* = 5 per group; *, *P* = 0.0008 [by *t* test]). Statistical bars depict the SEM. (H) Serum IL-6 from CASH and WASH treatments (*n* = 5 per group; *, *P* = 0.0135 [by *t* test]). Statistical bars depict the SEM. (I) Quantitative culture results on MacConkey plates: *, *P* = 0.003 (*n* = 5 per group [Mann-Whitney test]). (J) Percent mortality/dissemination across all experimental runs (*n* = 30, CASH treatment group; *n* = 40, WASH treatment group).

After 6 weeks on either a Western-type (W) or Chow (C) diet, mice were exposed to 5 days of antibiotic treatment with cefoxitin and clindamycin (A), preoperative starvation (S) (water only the night prior to surgery), and a 30% surgical hepatectomy (H) (i.e., CASH versus WASH) (Fig. 2A). A 30% hepatectomy is performed in <15 min and is a completely recoverable injury (14, 15). The morbidity score and survival curves demonstrated that the full complement WASH treatment was needed for morbidity and mortality to occur (Fig. 2B to F). Without antibiotic treatment (i.e., WSH or CSH), mice survived surgery with complete recovery to normal health status (Fig. 2B and C). Preoperative antibiotic treatment (cefoxitin, a broad-spectrum, second-generation cephalosporin, and clindamycin, a lincosamide antibiotic specific for anaerobes) significantly altered the postsurgical outcome which was highly dependent on the Western diet (Fig. 2D and E). In CASH-treated mice, only 2 of 15 animals developed a morbidity score of 5 (Fig. 2D). WASH-treated mice became moribund by 24 h, and 10 of 15 mice received the highest morbidity score of 5 at 40 h postoperatively (Fig. 2E). Kaplan-Meyer survival curves demonstrated 70% sepsis related mortality in WASH-treated mice (Fig. 2F).

CRP (Fig. 2G) and IL-6 (Fig. 2H) were significantly elevated in the blood of WASH-treated mice. This was associated with a high degree of dissemination of Gram-negative bacteria (10^5^ to 10^7^ CFU/g of tissue or ml of blood) (Fig. 2I). The dissemination of Gram-positive bacteria was negligible compared to Gram-negative bacteria (10^2^ CFU/g in tissues and sterile blood) in CASH-treated mice and absent in all samples from WASH-treated mice. When integrating all experimental runs and aggregated data, a low percentage of dissemination and mortality was observed in CASH-treated mice compared to a high percentages in WASH-treated mice (Fig. 2J).

**FIG 3:**
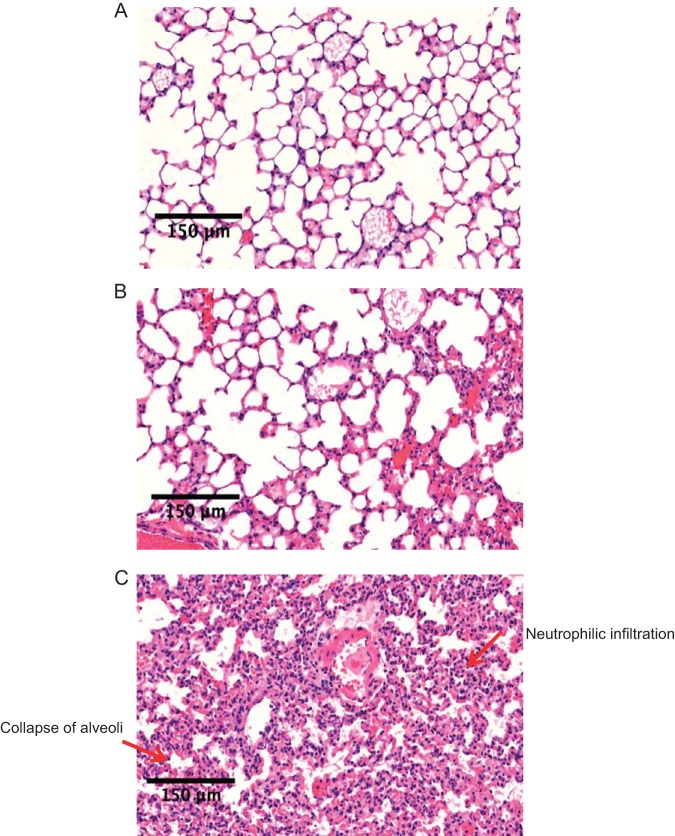
Effect of WASH treatment on organ damage. The lung histology of untreated (A), CASH-treated (B), and WASH-treated (C) mice is shown.

Representative histology of the lungs, the most common organ affected in animal models of sepsis, demonstrated heavy infiltration of inflammatory cells and collapse of alveoli in WASH-treated mice (Fig. 3C), which was markedly attenuated in the lungs of CASH-treated mice (Fig. 3B) and absent in normal untreated chow-fed mice (Fig. 3A).

### Domination by pathobionts in WASH-treated mice is associated with apoptosis at the bases of cecal crypts.

**FIG 4:**
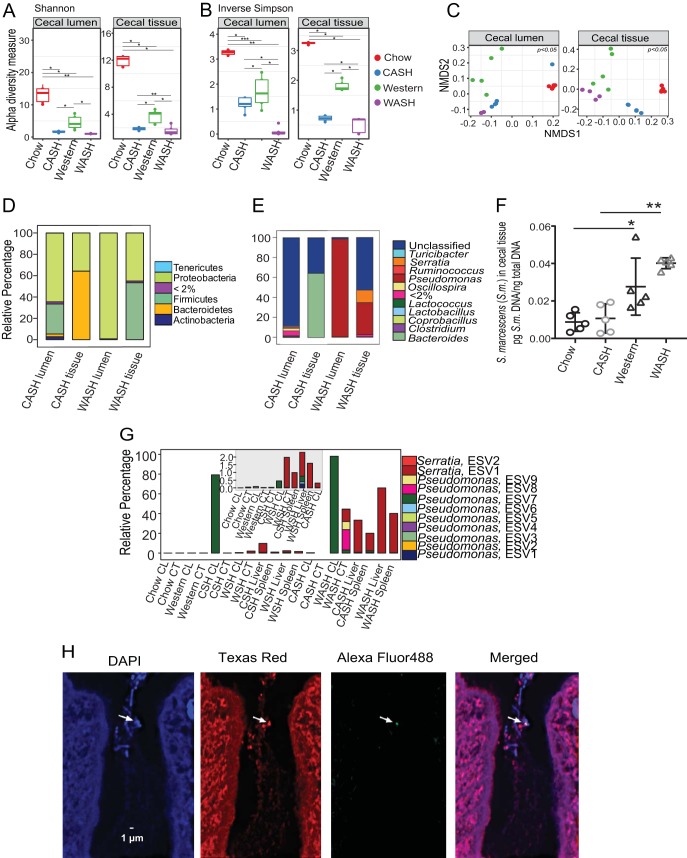
Comparative analysis of the cecal microbiota in CASH- and WASH-treated mice. (A and B) Alpha diversities based on Shannon (A) and invSimpson indexes (B). *n* = 5 mice per group; *, *P* < 0.05. (C) NMDS plots based on weighted UniFrac dissimilarity matrix: chow lumen versus CASH lumen (*P* = 0.002), chow tissue versus CASH tissue (*P* = 0.01), Western lumen versus WASH lumen (*P* = 0.03), Western tissue versus WASH tissue (*P* = 0.04), CASH lumen versus WASH lumen (*P* = 0.01), and CASH tissue versus WASH tissue (*P* = 0.03), evaluated using multigroup PERMANOVA. (D and E) Stack plots showing distribution of phyla (D) and genera (E) with relative abundances of >2%. A *P* value of <0.05 was determined with the Benjamini-Hochberg FDR correction in ANCOM. (F) qPCR analysis of cecal tissue-associated S. marcescens: Western versus chow (*, *P* = 0.048, *n* = 5 per group) and WASH versus CASH (**, *P* = 0.0005, *n* = 5 per group), determined using an unpaired *t* test with Welch’s corrections. (G) Distribution of ESVs belonging to the genera *Serratia* and *Pseudomonas*. The smaller inset shows the distribution of ESVs with low abundance (i.e., <2%). (H) FISH analysis of cecal tissue of untreated chow-fed mice indicates the presence of S. marcescens within a bacterial cluster inside a crypt (indicated by an arrow).

16S rRNA gene sequence analysis revealed a significant loss of alpha diversity in both lumen and tissue-associated microbiota on postoperative day 2 (POD2) independent of diet (Fig. 4A and B). Alpha diversity (Shannon diversity index) (Fig. 4A) decreased significantly in both WASH- and CASH-treated mice. By the inverse Simpson (Fig. 4B), diversity was also decreased in both groups but was significantly decreased in WASH-treated mice. Microbiota composition was also significantly altered on POD2 in both groups and across both compartments (cecal lumen and tissue-associated) based on the weighted UniFrac metric (Fig. 4C). The B:F:P ratio was shifted toward *Proteobacteria* in all groups: 1:10:30 in the lumen and 120:1:60 in the tissues in CASH-treated mice versus 0:0:100 in the lumens and 0:50:50 in the tissue-associated microbiota in WASH-treated mice (Fig. 4D). Notable, however, was the persistence of *Bacteroidetes* in the cecal tissues of CASH-treated mice but not in WASH-treated mice (Fig. 4D). At the genus level, most bacteria in the lumens of CASH-treated mice were unclassified; tissues from CASH-treated mice were dominated by *Bacteroides*; most bacteria in the lumens of WASH-treated mice were identified as *Pseudomonas*. In the tissues of WASH-treated mice *Pseudomonas* and *Serratia* dominated (Fig. 4E; see Table S2 in the supplemental material). To determine whether disseminated *Serratia* originated from the cecum, qPCR analysis of cecal tissues from Chow diet, WD, CASH-, and WASH-treated mice was performed. The results indicated the presence of Serratia marcescens DNA in all four groups, including untreated chow diet-fed mice (Fig. 4F). The cecal tissue S. marcescens DNA levels were significantly higher in WD-fed mice versus chow-fed mice, with the highest levels in WASH-treated mice (Fig. 4F). Using DeBlur and 16S rRNA amplicon sequence analysis, we determined the distinct organ distribution of exact sequence variants (ESVs) belonging to *Serratia* and *Pseudomonas*, two abundant genera in the cecal tissues of WASH-treated mice. We identified nine ESVs of *Pseudomonas*, among which, ESV7 was dominant in the cecal lumens of CSH- and WASH-treated mice. None of the *Pseudomonas* ESVs were dominant in extraintestinal organs (Fig. 4G). We also found two *Serratia* ESVs; among them, *Serratia* ESV2 was detected at very low abundance (0.05%) in the cecal tissue of untreated chow-fed mice. Notably, its abundance reached 2% following surgery in mice without preoperative antibiotic treatment and 30 to 60% in extraintestinal organs following antibiotic treatment and surgery (30% hepatectomy) (Fig. 4G). More abundant *Serratia* ESV2 was observed in livers and spleens in WASH-treated mice. To confirm the presence of S. marcescens in cecal tissues of nontreated mice, fluorescent *in situ* hybridization (FISH) was performed. S. marcescens was identified within bacterial clusters located at the bases of the cecal crypts (Fig. 4H), similar to what others have demonstrated for *Proteobacteria* (16).

10.1128/mBio.00903-19.6 TABLE S2 Relative abundances of the unclassified to genus-level ESVs in Fig. 4E. Download Table S2, XLSX file, 0.01 MB. Copyright © 2019 Hyoju et al. 2019 Hyoju et al. This content is distributed under the terms of the Creative Commons Attribution 4.0 International license.

**FIG 5:**
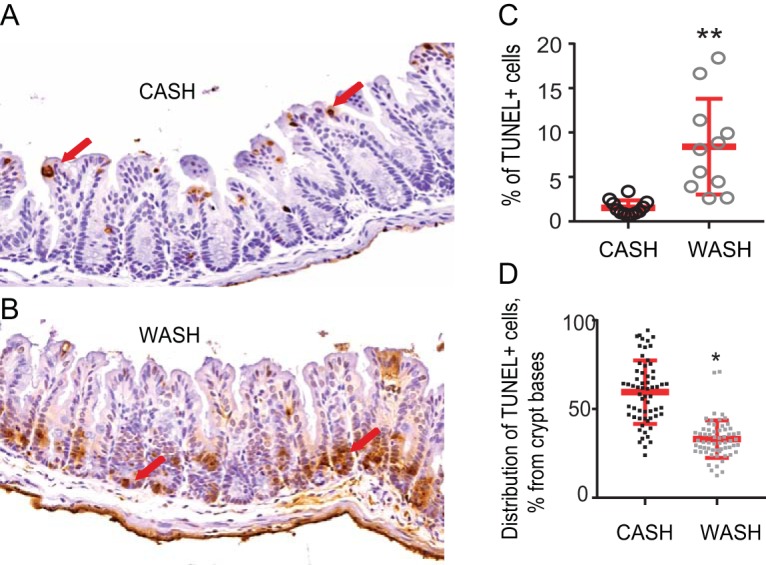
Effect of WASH treatment on cecal crypts. (A and B) Representative images demonstrating the abundance and distribution of TUNEL-positive cells in the cecal crypts of CASH-treated (A) and WASH-treated (B) mice. (C) Percent TUNEL-positive cells in cecal crypts (*n* = 3 mice [one-third of each tissue slide was counted separately]; **, *P* = 0.0017). (D) Percent distance of distribution of TUNEL-positive cells from the base to the top of the crypts (*n* = 3 mice [62 crypts with TUNEL-positive cells were counted per group]; *, *P* < 0.0001 [unpaired *t* test with Welch’s corrections]).

Next, we determined if the *Serratia* and *Pseudomonas* within tissue-associated microbiota has a significant effect on cecal crypt function using TUNEL (terminal deoxynucleotidyl transferase dUTP nick end labeling) staining (Fig. 5). Representative images indicated that apoptotic cells localize to the tops of crypts in CASH-treated mice (Fig. 5A), whereas they are concentrated at the base of cecal crypts in WASH-treated mice (Fig. 5B). Quantitative analysis demonstrated that the overall percentage of TUNEL-positive cells was significantly higher in the crypts of WASH-treated mice (Fig. 5C), with significantly larger amounts of TUNEL-positive cells in WASH-treated mice localized at the crypt base, the stem cell compartment (Fig. 5D). Apoptosis at the crypt base cells can lead to the loss of stem cells and impede the process of epithelial regeneration and recovery, resulting in irreversible damage to the tissues.

### WASH-treated mice harbor MDR strains of *Serratia marcescens* that express a virulent and immunosuppressive phenotype.

**FIG 6:**
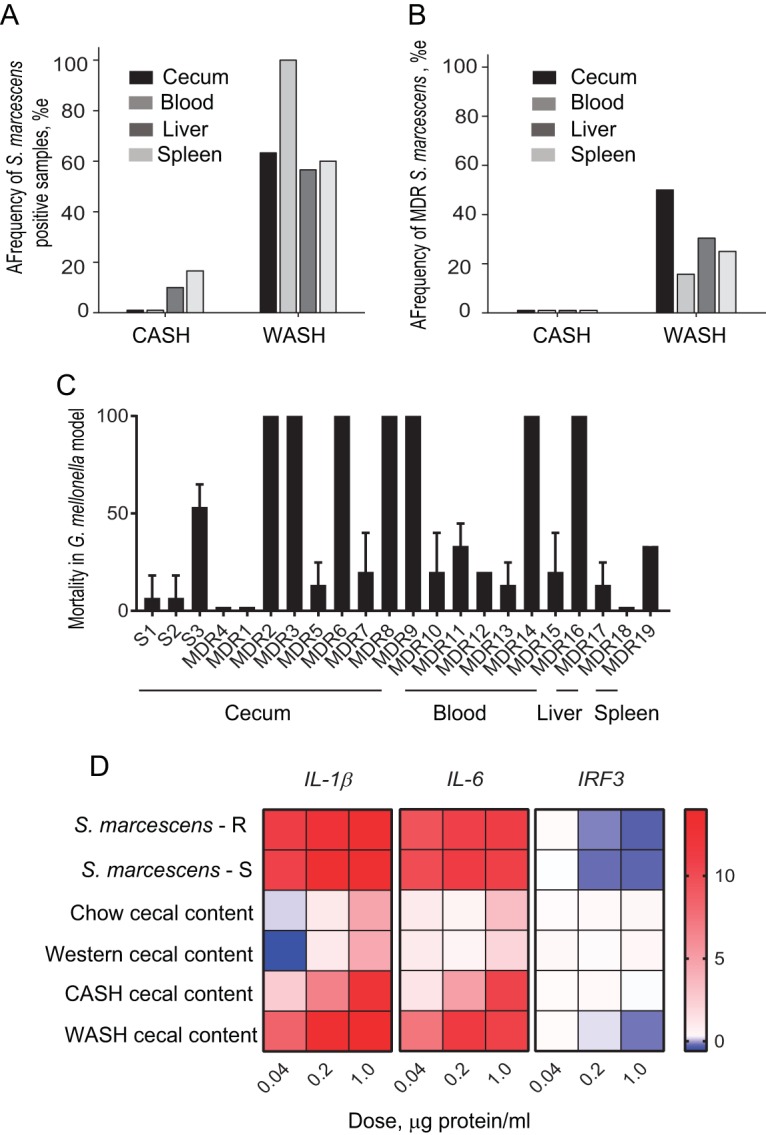
Quantitative and qualitative analysis of retrieved strains of Serratia marcescens. (A) Frequency of S. marcescens culture-positive organs in CASH and WASH treatment groups (*n* = 30 mice per group). (B) Frequency of S. marcescens MDR strains among isolates. (C) Virulence/killing assays using Galleria mellonella injected with selected antibiotic-sensitive and MDR strains of S. marcescens isolated from WASH-treated mice. Time of incubation after injection, 14 h (*n* = 45 larvae per bacterial strain [15 larvae × 3 biological replicates]). L, liver; S, spleen. (D) Heat maps representing the expression of genes encoding IL-1β, IL-6, and IRF3 in MEF cells in response to filtered lysates of S. marcescens and cecal contents from chow-fed, Western diet-fed, and CASH- and WASH-treated groups. The data are normalized to the expression of GAPDH (*n* = 3 per group). Error bars indicate the SEM.

Culture demonstrates that S. marcescens is the dominant species in the cecum, blood, liver, and spleen, with a higher prevalence in the WASH-treated mice versus CASH-treated mice (Fig. 6A). Among all S. marcescens isolates from WASH-treated mice, 10 to 50% express an MDR phenotype (Fig. 6B). MDR strains were defined based on their resistance to ≥3 classes of antibiotics. Extended-spectrum β-lactamase (ESBL)-producing and carbapenem-resistant strains of S. marcescens were flagged in the group of MDR strains. Further analysis of 21 randomly selected strains from this group revealed that 76% (16 of 21) of the MDR strains harbored an ESBL, and 95% (20 of 21) displayed resistance to meropenem (Table 1). None of the isolates, produced carbapenemase based on negative mCIM results. Importantly, no S. marcescens MDR strains were isolated in CASH-treated mice. We tested the virulence of MDR isolates using Galleria mellonella. Results demonstrated that among 19 MDR strains tested, 47% demonstrated a level of virulence similar to the three tested sensitive strains, and 36% of MDR strains displayed an extremely high virulence phenotype as judged by their ability to kill G. mellonella (Fig. 6C). The virulence of a given strain was not specific to the organ from which it was isolated. For example, of nine S. marcescens cecal isolates, two were found to be avirulent, and five were extremely virulent, as judged by G. mellonella killing assays.

**TABLE 1:** ESBL and mCIM test in group of MDR S. marcescens strains^*a*^

No.	Strain^*b*^	Source	Antibiotic resistance	ESBL	mCIM
1	T56447	WASH cecum	Cefepime^r^, ceftazidime^r^, ceftriaxone^r^, ciprofloxacin^i^, ertapenem^r^, gentamicin^r^, levofloxacin^i^, meropenem^r^, piperacillin^r^	POS	NEG
2	T23150	WASH blood	Cefepime^r^, ceftazidime^r^, ceftriaxone^r^, ciprofloxacin^r^, ertapenem^r^, gentamicin^r^, levofloxacin^r^, meropenem^r^, piperacillin^r^	POS	NEG
3	T23157	WASH blood	Cefepime^r^, ceftazidime^r^, ceftriaxone^r^, ciprofloxacin^r^, ertapenem^r^, gentamicin^r^, levofloxacin^r^, meropenem^r^, piperacillin^r^	NEG	NEG
4	T23147	WASH blood	Cefepime^r^, ceftazidime^r^, ceftriaxone^r^, ciprofloxacin^i^, ertapenem^r^, gentamicin^i^, levofloxacin^i^, meropenem^r^, piperacillin^r^	POS	NEG
5	T56439	WASH blood	Cefepime^r^, ceftazidime^r^, ceftriaxone^r^, ciprofloxacin^i^, ertapenem^r^, gentamicin^r^, levofloxacin^i^, meropenem^r^, piperacillin^r^	POS	NEG
6	T56441	WASH blood	Cefepime^r^, ceftazidime^r^, ceftriaxone^r^, ciprofloxacin^i^, ertapenem^r^, gentamicin^r^, levofloxacin^i^, meropenem^r^, piperacillin^r^	POS	NEG
7	T56443	WASH liver	Cefepime^r^, ceftazidime^r^, ceftriaxone^r^, ciprofloxacin^i^, ertapenem^r^, gentamicin^r^, levofloxacin^i^, meropenem^r^, piperacillin^r^	POS	NEG
8	T56444	WASH spleen	Cefepime^r^, ceftazidime^r^, ceftriaxone^r^, ciprofloxacin^i^, ertapenem^r^, gentamicin^r^, levofloxacin^i^, meropenem^r^, piperacillin^r^	POS	NEG
9	F75150 (13)	WASH cecum	Cefepime^r^, ceftazidime^r^, ceftriaxone^r^, ciprofloxacin^i^, ertapenem^r^, gentamicin^r^, levofloxacin^i^, meropenem^r^, piperacillin^r^	POS	NEG
10	F75151 (16)	WASH cecum	Cefepime^r^, ceftazidime^r^, ceftriaxone^r^, ciprofloxacin^i^, ertapenem^r^, gentamicin^r^, levofloxacin^i^, meropenem^r^, piperacillin^r^	NEG	NEG
11	F75152 (17)	WASH cecum	Cefepime^r^, ceftazidime^r^, ceftriaxone^r^, ciprofloxacin^i^, ertapenem^r^, gentamicin^r^, levofloxacin^i^, meropenem^r^, piperacillin^r^	POS	NEG
12	F75147 (8)	WASH cecum	Cefepime^r^, ceftazidime^r^, ceftriaxone^r^, ciprofloxacin^i^, ertapenem^r^, gentamicin^r^, levofloxacin^i^, meropenem^r^, piperacillin^r^	NEG	NEG
13	F75148 (9)	WASH cecum	Cefepime^r^, ceftazidime^r^, ceftriaxone^r^, ciprofloxacin^i^, ertapenem^r^, gentamicin^r^, levofloxacin^i^, meropenem^r^, piperacillin^r^	POS	NEG
14	F75149 (10)	WASH cecum	Cefepime^r^, ceftazidime^r^, ceftriaxone^r^, ciprofloxacin^i^, ertapenem^r^, gentamicin^r^, levofloxacin^i^, meropenem^r^, piperacillin^r^	POS	NEG
15	F75150 (12)	WASH cecum	Cefepime^r^, ceftazidime^r^, ceftriaxone^r^, ciprofloxacin^i^, ertapenem^r^, gentamicin^r^, levofloxacin^i^, meropenem^r^, piperacillin^r^	POS	NEG
16	T23208	WASH blood	Cefepime^r^, ceftazidime^r^, ceftriaxone^r^, ciprofloxacin^i^, ertapenem^r^, gentamicin^r^, levofloxacin^i^, meropenem^r^, piperacillin^r^	POS	NEG
17	T23213	WASH blood	Cefepime^r^, ceftazidime^r^, ceftriaxone^r^, ciprofloxacin^i^, ertapenem^r^, gentamicin^i^, levofloxacin^i^, meropenem^r^, piperacillin^r^	NEG	NEG
18	F75144 (4)	WASH cecum	Gentamicin^r^, piperacillin^r^, tobramycin^r^, trimethoprim/ sulfamethoxole^r^	NEG	NEG
19	T56430	WASH blood	Cefepime^r^, ceftazidime^r^, ceftriaxone^r^, ciprofloxacin^i^, ertapenem^r^, gentamicin^r^, levofloxacin^i^, meropenem^r^, piperacillin^r^	POS	NEG
20	T56432	WASH blood	Cefepime^r^, ceftazidime^r^, ceftriaxone^r^, ciprofloxacin^i^, ertapenem^r^, gentamicin^r^, levofloxacin^i^, meropenem^r^, piperacillin^r^	POS	NEG
21	T56434	WASH liver	Cefepime^r^, ceftazidime^r^, ceftriaxone^r^, ciprofloxacin^i^, ertapenem^r^, gentamicin^r^, levofloxacin^i^, meropenem^r^, piperacillin^r^	POS	NEG

a POS, positive; NEG, negative; ^r^, resistance; ^i^, intermediate resistance.

b Numbers in parentheses indicate no. of isolates.

To test the effect of S. marcescens on the innate immune response, we performed experiments using lysates of S. marcescens strains and cecal contents from chow-fed, WD-fed, and CASH- and WASH-treated mice. Lysates were applied onto mouse embryonic fibroblasts (MEFs) and immune responses tested *in vitro* (Fig. 6D) (17). Analysis by quantitative reverse transcription-PCR (qRT-PCR) after 6 h of coincubation was performed for genes encoding IL-1β and IL-6 and the gene encoding interferon regulatory factor 3 (IRF3), a key transcriptional regulator of type I interferons (IFNs) (18–20). The results demonstrated that S. marcescens, both multidrug-resistant and sensitive strains, upregulated the proinflammatory genes encoding IL-1β and IL-6 and downregulated the gene encoding IRF3. Higher concentrations of lysates were required for CASH to induce the same level of expression of proinflammatory genes, but IRF3 did not respond even at a high dose (1 μg of protein/ml) (Fig. 6D). Cecal lysates from chow-fed mice and mice fed a Western-type diet did not stimulate MEF cells (Fig. 6D). Given the downregulated response of IRF3 and its critical role in the immune response via type I IFNs, we performed a flow cytometry analysis of blood collected from CASH- and WASH-treated mice and compared it to chow-fed mice as a control. The results demonstrated a significant attenuation of B cells in both CASH- and WASH-treated mice, with a trend toward attenuation in WASH-treated mice and a significant decrease in naive and central memory CD4 T cells in WASH-treated mice (see Fig. S3 in the supplemental material). We also performed reiterative experiments comparing S. marcescens and E. faecalis, bacteria that were cultured from cecal tissue-associated microbiota. E. faecalis did not stimulate MEF cells to express proinflammatory genes (see Fig. S4 in the supplemental material). An additional bacterium isolated in this model, Enterobacter cloacae, stimulated MEF cells in a pattern similar to that for S. marcescens (see Fig. S4 in the supplemental material). This pathogen was isolated in several reiterative experiments in WASH-treated mice in which mortality was not associated with S. marcescens, but rather E. cloacae disseminated in the blood, liver, and spleen. In this experiment, similar to S. marcescens, E. cloacae expressed the MDR phenotype but did not produce ESBL or carbapenemase enzymes.

10.1128/mBio.00903-19.3 FIG S3 Adaptive immune responses as analyzed by flow cytometry from blood samples. (A and A′) Flow plots (A) and estimation (A′) of population of B cells (CD19^+^). (B and C) Naive CD44^−^ CD62L^+^ (B) and central memory CD44^+^ CD62L^+^ (C) subsets of CD4 T cells. Download FIG S3, PDF file, 0.1 MB. Copyright © 2019 Hyoju et al. 2019 Hyoju et al. This content is distributed under the terms of the Creative Commons Attribution 4.0 International license.

10.1128/mBio.00903-19.4 FIG S4 Heat maps of genes encoding IL-1β, IL-6, and IRF3 of MEFs exposed to filtered bacterial lysates. Enterobacter cloacae (E.c.), Enterococcus faecalis (*E.f*.), S. marcescens MDR (*S.m*.-R), and antibiotic sensitive (*S.m*.-S) isolates, as well as cecal contents from the chow, WD, CASH, and WASH groups. Data are normalized to the expression of GAPDH (*n* = 3 per group). MEFs were incubated for 6 and 12 h with lysates, and cecal contents were adjusted to optical density of 0.2. Download FIG S4, PDF file, 0.1 MB. Copyright © 2019 Hyoju et al. 2019 Hyoju et al. This content is distributed under the terms of the Creative Commons Attribution 4.0 International license.

## DISCUSSION

By combining the effects of an obesogenic diet and antibiotic exposure, we observed spontaneous emergence and dissemination of pathobionts associated with organ damage and lethality following an otherwise recoverable surgical injury. Surprisingly, among the isolated pathogens, ∼20% displayed an MDR phenotype that appeared unrelated to the antibiotics used in this model. The results from the present study support the observations in humans that MDR pathogens causing bacteremia in patients may emerge from the intestinal microbiome rather than from a grossly infected organ or direct environmental contamination (6, 7, 21).

Surgery was required for mortality to occur in this model. Not only is the physiologic stress of surgical injury known to cause collapse of microbiota community structure and function (22, 23), but it is also known to alter intestinal barrier function and accelerate intestinal apoptosis, two interrelated events (24). However, intestinal barrier function alterations following surgical injury alone (such as burn, trauma, or hemorrhagic shock) rarely if ever lead to lethality perhaps owing to the fact that the organisms that translocate under these circumstances are mainly commensal Escherichia coli, which translocate at low bacterial density and which themselves are not lethal pathogens (23, 25).

Interestingly, the species of pathobionts that emerged in the current model, S. marcescens and E. cloacae, are often those found in ultralow-diversity communities in critically ill patients with organ failure and sepsis (26). The difficulty in detecting these pathogens preoperatively may be due to their being camouflaged within hidden niches of the gut, such as the cecal crypts (16). *Bacteroides* persisted in the cecal mucosa of CASH-treated mice and may have played a role in preventing mortality via its ability to produce short-chain fatty acids, suppress pathobionts even in the face of antibiotics and surgery, and its ability to preserve the mucosal mucus layer and barrier function (27).

In cases of both human and experimental sepsis, bacteremia is both rare and highly variable (28–30). Less than one-third of patients with severe sepsis are found to have bacteria in their blood either by culture or PCR (28–30). We were able to detect, among WASH-treated mice, significant bacteremia and culture positivity in the liver, spleen, and blood compared to culture negativity across all sites among the other treatment groups (CASH, CSH, and WSH). Although causality between bacterial dissemination and mortality was not established in this model, the ability to capture and characterize MDR S. marcescens and others in WASH-treated mice suggests that multiple perturbations to the intestinal microbiota, including diet, antibiotic exposure, and surgery, are needed for this to occur, although it does not occur in all WASH-treated mice. The exact mechanism by which S. marcescens developed resistance to carbapenem remains to be determined, but, given the lack of carbapenemase production, we hypothesized that this observed carbapenem resistance was likely due to the hyperproduction of AmpC β-lactamase (31). There are several potential explanations for how MDR strains might have emerged in this model. First, they may have been introduced into the mouse gut from the local environment (housing facility, during transportation, etc.). Second, they may have been part of the normal flora and were selected for by the conditions of WASH treatment. Finally, it is possible that commensal strains were transformed to MDR strains by effects of WASH treatment on their evolutionary trajectory. The latter possibility is supported by existing evidence demonstrating that microbes can become antibiotic resistant without being exposed to the specific antibiotics that confer resistance (32). Testing this possibility will require serial genetic tracking of the intestinal microbiota at the strain level, as well as ultradeep sequencing. In addition, there is evidence that antibiotic-resistant strains are normal members of the intestinal microbiome (33). Also relevant to the present study is the observation that obese patients are more likely to harbor bacterial resistance genes (34) and are also more susceptible to postoperative infections (35).

Potential mechanisms of any of the above possibilities may relate, in part, to the known increase in bile acid synthesis observed in obese hosts (36). The potent antimicrobial activity of bile acids has been shown to directly regulate the gut microbiota (37, 38), including effects such as the disruption of bacterial membranes, the denaturation of proteins, the chelation of iron and calcium, the oxidative damage of DNA, etc. (39). Furthermore, a Western-type diet has been demonstrated to increase oxidative stress (40, 41), which can also impact the emergence of antibiotic resistance (42–44). Finally, it is possible that WASH treatment eliminates epithelial protective *Bacteroidetes* and other microbiota that competitively suppress the pathobionts. Without *Bacteroides* and other cytoprotective microbiota, enteric pathogens can disseminate, as observed here in the WASH-treated mice (Fig. 2D).

A limitation of this study is that the precise compositional elements responsible for the effect of diet on the composition of gut microbiota in this model are unknown since the two diets varied significantly in fatty acids, fiber content, carbohydrates, minerals, and vitamins. Any number of combinations of these nutrients could potentially influence the observed decrease in *Bacteroidetes* (45, 46) and *Actinobacteria* (47, 48) and the bloom of *Proteobacteria* (49, 50). Now that the model is fully characterized up to the endpoint of organ damage and lethality, reiterative studies using various single nutrients and related groups of nutrients can be carried out. Similarly, changing the type and duration of antibiotic exposure, the environmental factors (sleep, social stress, etc.), the background microbiota, and the mouse genetics is now possible in this model. Another limitation is that mice were not housed separately for 6 weeks after randomization to the specific diets, which may have introduced a “cage effect” on the microbiota. However, given the multiple experimental runs, the reproducibility of the impact of diet on the microbiota, and the large sample size, the direct influence of the cage effect on our results is likely to have been minimized.

In summary, we present here a novel model of lethal sepsis occurring without the introduction of an exogenous pathogen and without creating a gross untreated infection such as occurs with the cecal ligation and puncture model. The model described here may advance our understanding of the mortality of human sepsis, which most often involves MDR pathogens, immunosuppression, and the absence of an active untreated infectious focus.

## MATERIALS AND METHODS

### Animals.

Male C56BL/6 mice (Charles River laboratory), 6 weeks old, with an average weight of 22 g (range, 20 to 24 g) were used. The mice were housed in a temperature-controlled 12-h light/dark cycled room at the University of Chicago animal facility for 1 week. Mice were randomly assigned to *ad libitum* feeding of either standard mouse chow (Envigo) or a Western-type diet (Bio Serve, mouse high-fat diet, catalog no. S3282) (see Table S1 in the supplemental material). To avoid social isolation stress, mice were housed at five mice per cage for 6 weeks after random assignment to their respective diets. Mice were weighed every 3 days. All experiments were performed in accordance with the National Institutes of Health (NIH) guidelines, and approval was obtained from the University of Chicago Animal Care and Use Committee (protocol 71744).

10.1128/mBio.00903-19.5 TABLE S1 Comparative analysis of ingredients in chow and Western diet. Download Table S1, DOCX file, 0.01 MB. Copyright © 2019 Hyoju et al. 2019 Hyoju et al. This content is distributed under the terms of the Creative Commons Attribution 4.0 International license.

### Surgical procedure.

Following dietary allocation, antibiotics were administered as a combination of cefoxitin and clindamycin. Cefoxitin (30 mg/kg; Hikma Pharmaceutical, Eatontown, NJ) was administered subcutaneously, and clindamycin (70 mg/kg; Clindrops; Henry Schein, Dublin, OH) was administered by gavage twice daily for 5 days before surgery. Five days of antibiotic exposure was necessary for sepsis/mortality to develop in this model (data not shown). Mice were individually housed and only given water for 14 h prior to surgery. Mice were anesthetized by intraperitoneal injection of ketamine at 80 mg/kg and xylazine at 10 mg/kg, with injection of buprenorphine at 0.05 at mg/kg 30 min before anesthesia. The abdomen was sterilely prepared and left lobe of liver excised bloodlessly. For recovery, mice received a subcutaneous injection of 1 ml of warm saline (37°C) and buprenorphine at 8 h. Mice were assessed every 6 h according to standard sepsis scoring system: score 1, ambulatory, active, normal fur coat, normal amount of feces (normal); score 2, ambulatory, active, ruffled fur (mild sepsis); score 3, ruffled fur, hunched posture, increased respirations (moderate sepsis); score 4, ruffled fur, hunched posture, increased respirations, slowly staggering gait in response to touch (severe sepsis); and score 5, animal on side, minimally responsive, rapid shallow respirations, gasping (moribund).

### Sample collection.

Blood was collected aseptically by direct cardiac puncture. A portion of the liver and spleen was homogenized for culture. Cecal tissue and content were collected in 10% glycerol for culture and frozen for DNA extraction. Finally, lung and cecal tissue were fixed in formalin for histology.

### Bacterial culture.

Portions (100 μl) of blood, liver, and spleen homogenates were serially diluted and plated on MacConkey and Columbia nalidixic acid agar plates (BD Difco), and the CFU counted at 24 h.

### IL-6 and CRP serum assays.

CRP and IL-6 concentrations were assayed using commercially available kits (BD OptEIA; R&D Systems, Minneapolis, MN).

### 16S rRNA sequencing and sequence data analysis.

Microbial DNA extraction from cecal contents was performed using a PowerSoil DNA isolation kit (MoBio, Carlsbad, CA). Tissues were homogenized, and DNA was extracted using an UltraClean tissue and cells DNA isolation kit (MoBio). For library preparation, DNA was amplified using the barcoded 12-bp Golay primer set designed for the Earth Microbiome Project (EMP) (51). PNAClamp technology (PNA Bio) was used to prevent the amplification of the murine 16S mitochondrial region. For each sample, PCR was performed according to the manufacturer’s protocol using the EMP primers, mPNA, AccuStart II PCR ToughMix, and the extracted DNA (Quntabio). Since the PNA requires a hold at 75°C for 10 s in order to “clamp” onto contaminant DNA, the standard EMP 16S PCR schedule was modified to the following: 94 for 3 min; 35 cycles of 94 for 45 s, 75 for 10 s, 50 for 1 min, and 72 for 1.5 min; and a final extension at 72 for 10 min. After amplification, the PCR products were quantified by using a PicoGreen dsDNA quantitation assay (Invitrogen). The results of the quantification were then used to normalize the amount of DNA from the PCR product to use for sequencing and to ensure that each amplicon was represented evenly during sequencing. These volumes were then sequentially consolidated into a single tube using an OpenTrons liquid-handler running a custom Python script. Finally, an aliquot of the final pool was taken, and the DNA was purified by using an Agencourt AMPure XP PCR* purification system (Beckman-Coulter). The samples were then run on an Illumina MiSeq at the University of Illinois at Chicago (150 bp × 2). The data are available in EMBL-ENA under BioProject ID PRJEB32897 (secondary accession no. ERP115632), along with the metadata.

For 16S rRNA gene sequence analysis, the paired-end reads were joined using the join_paired_ends.py script, followed by quality filtering and demultiplexing using the split_libraries_fastq.py script in QIIME (52). The final set of demultiplexed sequences was then selected for ESV picking using the DeBlur pipeline (53). To improve downstream network analysis, ESVs present in ≤10 samples were also removed. The data were also rarefied to a depth of 10,000 reads per sample. Alpha and beta diversities were analyzed using the Phyloseq and MicrobiomeSeq package in R. For the alpha diversity, the Shannon index was used, and the beta diversity was analyzed using nonmetric multidimensional scaling (NMDS) plots that were generated based on a weighted UniFrac dissimilarity matrix. Assessment of statistical significance of alpha and beta diversity trends was performed using permutational analysis of variance (PERMANOVA). To determine significantly different ESVs between groups of interest, an analysis of composition of microbiome (ANCOM) pipeline was used at *P* value (false discovery rate [FDR]) cutoff of <0.05.

### TUNEL analysis.

TUNEL staining was performed using an ApopTag Plus Peroxidase In Situ apoptosis detection kit (Millipore) as previously described (54). Determination of the TUNEL-positive cell fraction was performed by color deconvolution of scanned gut images, followed by setting the threshold to a positive signal and determination of the area fraction by using built-in Fiji plugins. The distribution of TUNEL cells per villus was automated using a custom macro script in Fiji. Villi were traced with a wide line region of interest, the villus was straightened and rotated to be horizontal with the base on the left, the positive cells were identified using a manually confirmed threshold, and the distance to each cell centroid was determined relative to the villus tip using standard Fiji plugins. Aligned villi were assembled into a common (minimum) projection to pictorially show the location of cells along the villi.

### FISH staining.

FISH staining for visualization of S. marcescens was performed as previously described (54) using a probe S. marcescens Alexa Fluor488 designed by MetaSystems Indigo GmbH, Altusscheim, Germany, which uses a next generation FISH beacon-based technology. A bacterial universal Texas red probe was applied for dual staining. Confocal microscopy was performed on a Leica SP5 II AOBS tandem scanner spectral confocal system on a DMI6000 microscope and controlled by LASAF software (v2.8.3).

### MEF experiments.

C57BL/6 primary MEFs were obtained from 12.5 to 14.5 days postcoitus embryos and cultured in Dulbecco modified Eagle medium (Gibco) supplemented with 10% fetal bovine serum (Gibco) and 1% nonessential amino acids (Gibco). MEFs were plated at a density of 2.5 × 10^5^/ml in 24-well plates (Costar) overnight. Pathogen lysates were prepared by taking live pathogens in culture, resuspension in PBS, bead beating with 0.1-mm-diameter glass beads (BioSpec) for 5 min, and filtration using a Millex-GV 0.22-μm-pore-size filter (Millipore). Cecal content lysates were prepared by bead beating cecal contents with 0.1-mm-diameter glass beads (BioSpec) for 5 min and filtration using a Millex-GV 0.22-μm-pore-size filter (Millipore). Protein concentrations were measured by using a BCA protein assay kit (Thermo Fisher Scientific). Plates were incubated at 37°C and 5% CO_2_ for 6 or 12 h with the addition of lysates; the culture medium was then removed, and the cells were harvested in RLT Plus buffer (Qiagen) with 1% 2-mercaptoethanol (Sigma). RNA extraction was performed using an RNeasy Plus minikit (Qiagen) according to the manufacturer’s instructions. Two independent experiments were performed, with technical replicates.

### Transcriptional analysis of MEFs using qRT-PCR assay.

RNA was converted to cDNA using reverse transcriptase (Promega) according to the manufacturer’s instructions. Samples were run in duplicate via qPCR on a Roche LightCycler 480 using SYBR Advantage qPCR Premix (Clontech). The expression levels were quantified and normalized to housekeeping gene GAPDH (glyceraldehyde-3-phosphate dehydrogenase). The following qPCR primer pairs were custom ordered from Integrated DNA Technologies: IL1β-F (GAAATGCCACCTTTTGACAGTG) and IL1β-R (TGGATGCTCTCATCAGGACAG); IL-6-F (CCAAGAGGTGAGTGCTTCCC) and IL-6-R (CTGTTGTTCAGACTCTCTCCCT); IRF3-F (GAGAGCCGAACGAGGTTCAG) and IRF3-R (CTTCCAGGTTGACACGTCCG); and GAPDH-F (AGGTCGGTGTGAACGGATTTG) and GAPDH-R (TGTAGACCATGTAGTTGAGGTCA).

### Microbial phenotype microarray.

The GEN III MicroPlate test panel (Biolog, Hayward, CA) was used to determine the phenotypic fingerprint of microbial community in chow-fed, WD-fed, CASH-, and WASH-treated mice. Cecal contents were collected in saline with 10% glycerol and in saline with 10% glycerol and 1% cysteine (to preserve anaerobic bacteria) at the time of sacrifice and stored at –80°C until use. Samples were centrifuged at 100 rpm for 1 min to remove tissue debris, and the bacterial cell suspension was diluted in saline to an optical density at 600 nm of 0.25. Then, 50 μl of the sample was introduced in the IF-A inoculation fluid vial (Biolog). Each diluted sample was placed on 96-well GENIII microplates (Biolog), followed by incubation for 22 h at 37°C in an OmniLog incubator/reader (Biolog). Processing of anaerobic samples was performed in anaerobic chamber, and the plates were sealed with Microseal B seal (Bio-Rad). Data analysis was performed with the statistical program R and the “opm” package. Growth curve parameters (area under the concentration-time curve [AUC] and maximum) were aggregated from the data using the “do_aggr” function. The data were normalized to the AUC of the negative control, since this was the well on the plate without substrate to affect the growth of the microbial community in the samples. Heat maps and radial plots were created using the “heat_map” and “radial_plot” functions. Pairwise comparisons (analysis of variance) between the groups was performed with the “opm_mcp” function.

### Bacterial identification and antibiotic resistance profiling.

Bacterial identification was performed on the Vitek MS (bioMérieux, Inc., Durham, NC). Susceptibility testing of Gram-negative bacilli was performed using the Vitek 2 XL system (bioMérieux) using the AST-GN75 card. Confirmatory testing for ESBL production was performed on Mueller-Hinton agar using cefotaxime and ceftazidime disks, with or without clavulanic acid (BD, Franklin Lakes, NJ). A difference of ≥5 mm in zone size between the clavulanic acid-containing disks and the disks without clavulanic acid was considered positive for ESBL production. The modified carbapenem inactivation method (mCIM) was performed to detect carbapenemase production. The test was performed as described in the 28th edition of the Clinical and Laboratory Standards Institute supplement M100 (55).

### Statistical analysis.

Data were analyzed using Prism (GraphPad Software, San Diego, CA). The results were expressed as means ± the standard errors of the means (SEM). Nonparametric Mann-Whitney, one-way ANOVA test, and the log rank test (Mantel-Cox test) were used to test the statistical significance. A *P* value of <0.05 was considered significant. The statistical program R and the “opm” package provided by Biolog were used to analyze phenotypic microarray data.
