# Supplementary material for: Mice Fed an Obesogenic Western Diet, Administered Antibiotics, and Subjected to a Sterile Surgical Procedure Develop Lethal Septicemia with Multidrug-Resistant Pathobionts
Source: mBio. 2019 Jul 30;10(4):e00903-19. doi: 10.1128/mBio.00903-19 (PMC6667615; doi:10.1128/mBio.00903-19)
Supplement: FIG S1 [file mBio.00903-19-sf001.pdf]

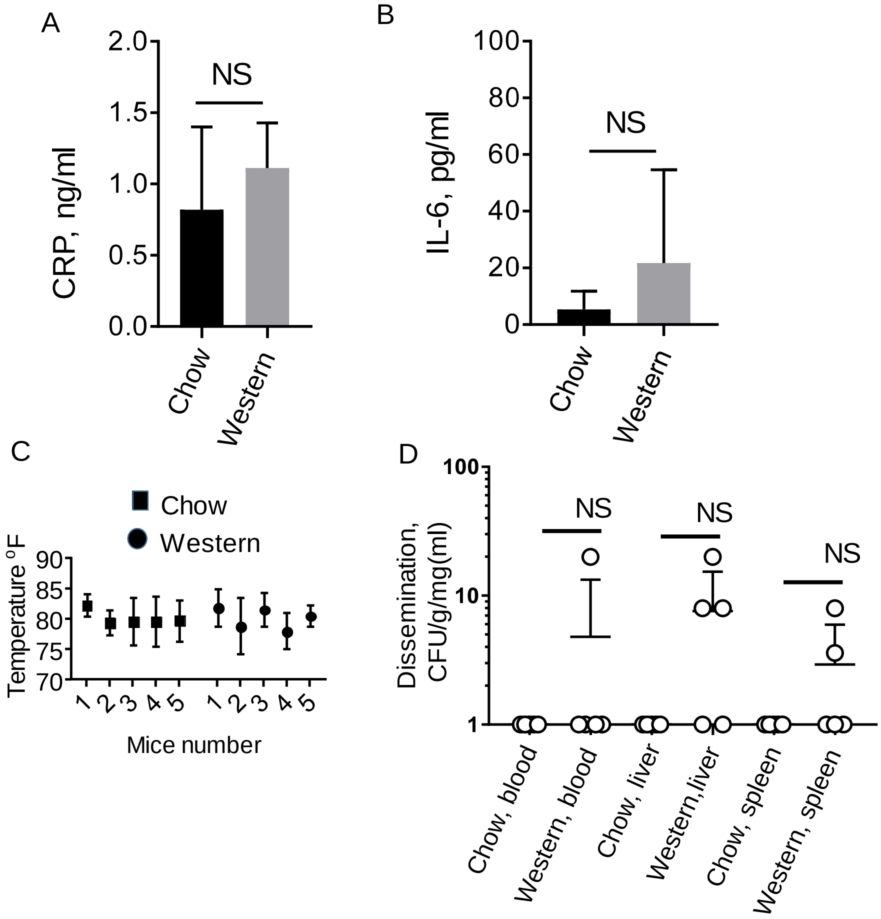

Fig.A1. Analysis of sepsis markers in Western- and Chow- fed mice measured before antibiotic treatment. (A), C-reactive protein (CRP. p=NS, n=4 per group. (B), Serum IL-6 levels pg/ml, p=NS, n=4 per group. (C), Body surface temperature measured by laser instrument reflecting minimum and maximum measurements. p=NS. n= 5 mice per group. (D), Colonization of blood, liver and spleen by gram-negative bacteria on MacConkey plates. p=NS, n=5 per group, Mann-Whitney test.
