# Supplementary material for: Mice Fed an Obesogenic Western Diet, Administered Antibiotics, and Subjected to a Sterile Surgical Procedure Develop Lethal Septicemia with Multidrug-Resistant Pathobionts
Source: mBio. 2019 Jul 30;10(4):e00903-19. doi: 10.1128/mBio.00903-19 (PMC6667615; doi:10.1128/mBio.00903-19)
Supplement: FIG S2 [file mBio.00903-19-sf002.pdf]

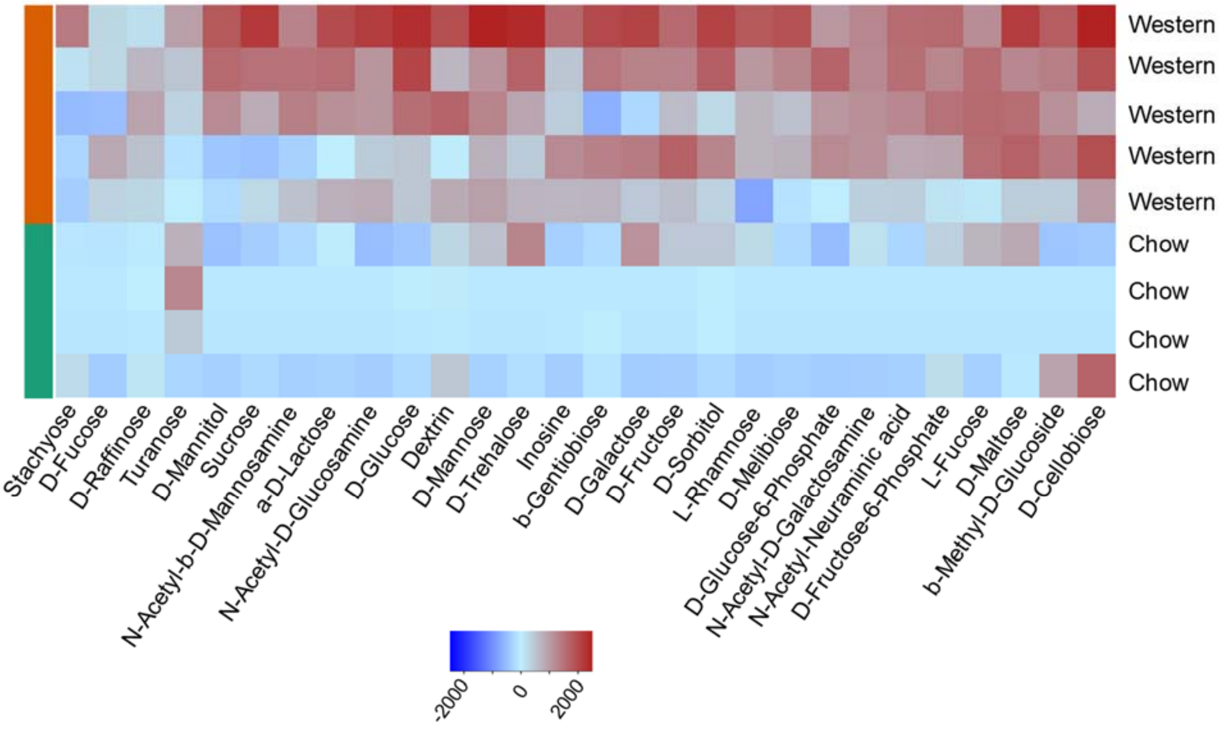

Fig.A2. Comparative carbohydrate metabolic analysis of cecal contents isolated from Western- and Chow-fed mice. Analysis using GENIII plates to assess microbial phenotype.
