# Supplementary material for: Mice Fed an Obesogenic Western Diet, Administered Antibiotics, and Subjected to a Sterile Surgical Procedure Develop Lethal Septicemia with Multidrug-Resistant Pathobionts
Source: mBio. 2019 Jul 30;10(4):e00903-19. doi: 10.1128/mBio.00903-19 (PMC6667615; doi:10.1128/mBio.00903-19)
Supplement: FIG S3 [file mBio.00903-19-sf003.pdf]

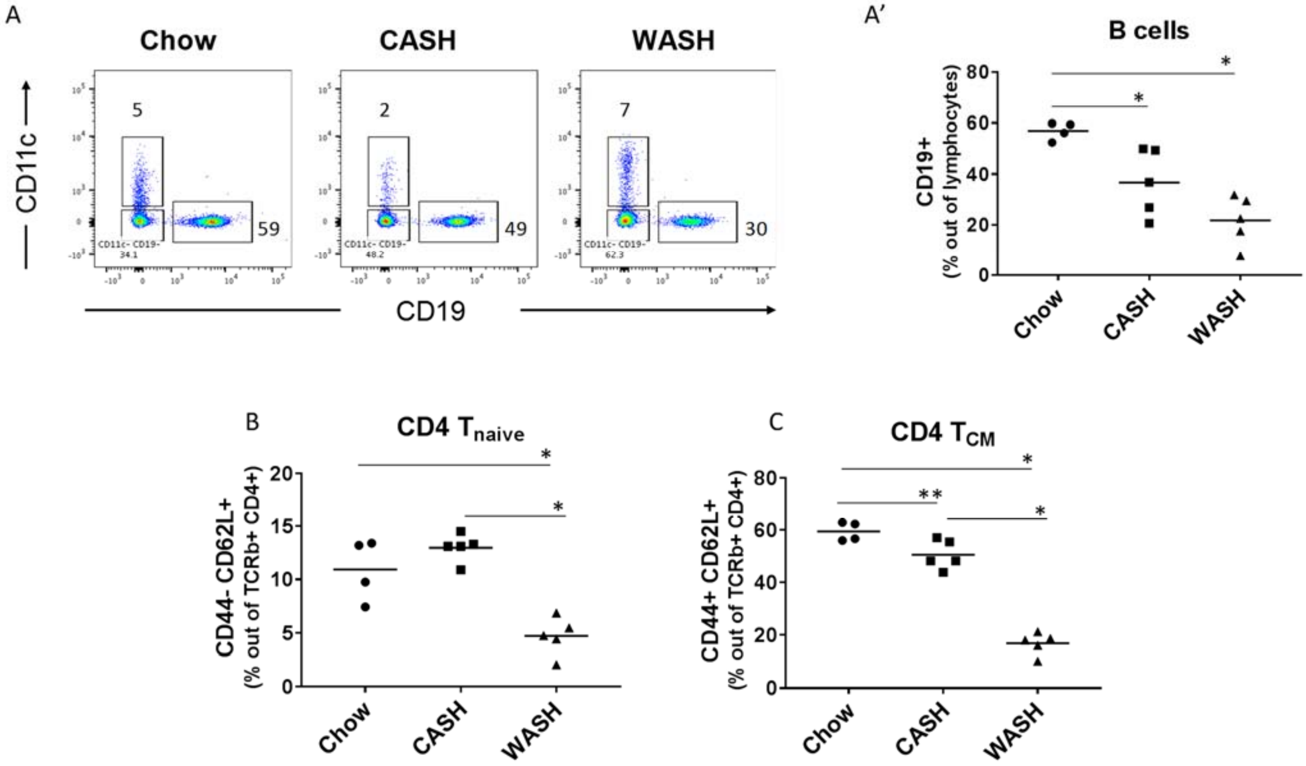

Fig. A3. Adaptive immune responses as analyzed by flow cytometry from blood samples. (A, A'), Flow plots (A) and estimation (A') of population of B cells ($CD19^{+}$). (B, C), naïve $CD44^{-}$ $CD62L^{+}$ (B) and central memory $CD44^{+}CD62L^{+}$ (C) subsets of CD4 T cells.
