# Supplementary material for: Mice Fed an Obesogenic Western Diet, Administered Antibiotics, and Subjected to a Sterile Surgical Procedure Develop Lethal Septicemia with Multidrug-Resistant Pathobionts
Source: mBio. 2019 Jul 30;10(4):e00903-19. doi: 10.1128/mBio.00903-19 (PMC6667615; doi:10.1128/mBio.00903-19)
Supplement: FIG S4 [file mBio.00903-19-sf004.pdf]

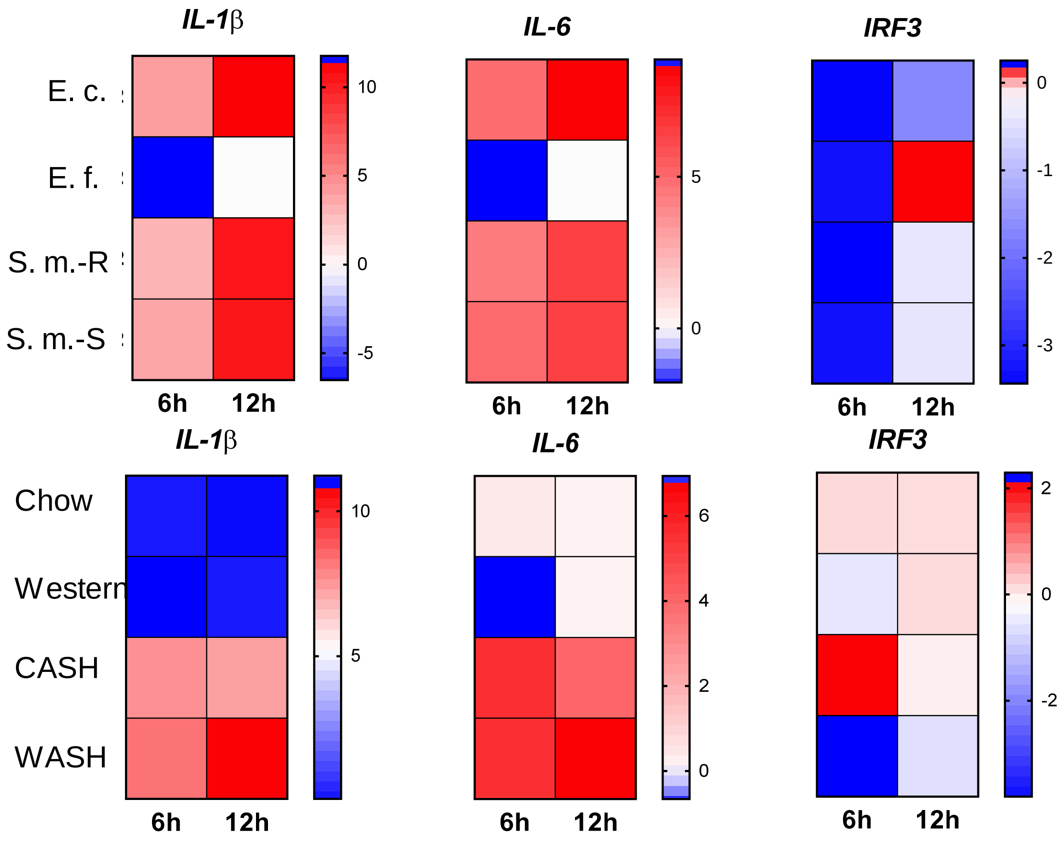

Fig.A4. Heat maps of genes encoding IL-1β, IL-6, and IRF3 of mouse embryonic fibroblasts (MEF) exposed to filtered bacterial lysates. *Enterobacter cloacae* (E.c.), *Enterococcus faecalis* (E.f.), *S. marcescens* MDR (S.m.-R) and antibiotic sensitive (S.m.-S) isolates, as well as cecal contents from Chow, Western, CASH, and WASH groups. Data normalized to the expression of GAPDH. n=3 per group. MEF cells were incubated for 6 and 12 hours with lysates and cecal contents adjusted to OD 0.2.
