# Supplementary material for: Mice Fed an Obesogenic Western Diet, Administered Antibiotics, and Subjected to a Sterile Surgical Procedure Develop Lethal Septicemia with Multidrug-Resistant Pathobionts
Source: mBio. 2019 Jul 30;10(4):e00903-19. doi: 10.1128/mBio.00903-19 (PMC6667615; doi:10.1128/mBio.00903-19)
Supplement: TABLE S1 [file mBio.00903-19-st001.docx]

| **##** | **Ingredients** | **Chow** | **Western** | **##** | **Ingredients** | **Chow** | **Western** |
| --- | --- | --- | --- | --- | --- | --- | --- |
| **1. Fatty acids, gm/kg** | Total saturated | 9 | 141 | **10.** | **Vitamins, mg/kg** |  |  |
|  | Total monounsaturated | 13 | 162 |  | Vitamin K3 (menadione) | 50 | 0.52 |
|  | Total polyunsaturated | 34 | 40.2 |  | Vitamin B1 (thiamin) | 17 | 3 |
|  |  |  |  |  | Vitamin B2 (riboflavin) | 15 | 2.3 |
| **2. Fat, %** |  | 6.2 | 36 |  | Niacin (nicotinic acid) | 70 | 15 |
| **3. Proteins, %** |  | 18.6 | 20 |  | Vitamin B6 (pyridoxine) | 18 | 4.1 |
| **4. Fiber, %** | Crude fiber | 3.5 | 0 |  | Pantothenic Acid | 33 | 5.5 |
|  | Neutral detergent fiber | 14.7 | 0 |  | Vitamin B12 (cyanocobalamin) | 0.08 | 0.04 |
|  |  |  |  |  | Folate | 4 | 0.75 |
| **5.** **Crude protein, %** |  | 18.6 | 20.5 |  | Choline | 1200 | 1148 |
| **6.** **Carbohydrates, %** | Simple sugars | 3 | 22 |  |  |  |  |
|  | Complex sugars | 41 | 14 | **11.** | **Vitamins, IU/kg** |  |  |
| **7. Calories, kcal/gm** | Calories from protein | 0.24 | 0.82 |  | Vitamin A | 15000 | 3162 |
|  | Calories from fat | 0.18 | 3.24 |  | Vitamin E | 110 | 25.7 |
|  | Calories from carbohydrate | 0.58 | 1.43 |  |  |  |  |
| **8. Minerals, gm/kg** | Calcium | 10 | 5.6 |  |  |  |  |
|  | Chloride | 4 | 0.86 |  |  |  |  |
|  | Phosphorus | 7 | 5.8 |  |  |  |  |
|  | Sodium | 2 | 0.57 |  |  |  |  |
|  | Potassium | 6 | 5.6 |  |  |  |  |
|  | Magnesium | 2 | 0.49 |  |  |  |  |
|  | Zinc | 0.07 | 0.022 |  |  |  |  |
|  | Manganese | 0.1 | 0.047 |  |  |  |  |
|  | Copper | 0.015 | 0.004 |  |  |  |  |
|  | Iodine | 0.006 | 0.003 |  |  |  |  |
|  | Iron | 0.2 | 0.05 |  |  |  |  |
| **9. Amino Acids, gm/kg** | Aspartic Acid | 14 | 12.8 |  |  |  |  |
|  | Glutamic Acid | 34 | 40.6 |  |  |  |  |
|  | Alanine | 11 | 5.3 |  |  |  |  |
|  | Glycine | 8 | 4.9 |  |  |  |  |
|  | Threonine | 7 | 8.7 |  |  |  |  |
|  | Proline | 16 | 20.5 |  |  |  |  |
|  | Serine | 11 | 11.4 |  |  |  |  |
|  | Leucine | 18 | 16.6 |  |  |  |  |
|  | Isoleucine | 8 | 11 |  |  |  |  |
|  | Valine | 9 | 13 |  |  |  |  |
|  | Phenylalanine | 10 | 8.9 |  |  |  |  |
|  | Tyrosine | 6 | 11.4 |  |  |  |  |
|  | Methionine | 4 | 7.1 |  |  |  |  |
|  | Cystine | **3** | 0.6 |  |  |  |  |
|  | Lysine | 9 | 14.8 |  |  |  |  |
|  | Histidine | 4 | 5.5 |  |  |  |  |
|  | Arginine | 10 | 7.3 |  |  |  |  |
|  | Tryptophan | 2 | 2.2 |  |  |  |  |
|  |  |  |  |  |  |  |  |

**Table A1. Comparative analysis of ingredients in Chow and Western diet**
